# Lateral root formation and growth of *Arabidopsis* is redundantly regulated by cytokinin metabolism and signalling genes

**DOI:** 10.1093/jxb/ert291

**Published:** 2013-09-10

**Authors:** Ling Chang, Eswarayya Ramireddy, Thomas Schmülling

**Affiliations:** Institute of Biology/Applied Genetics, Dahlem Centre of Plant Sciences (DCPS), Freie Universität Berlin, Albrecht-Thaer-Weg 6, D-14195 Berlin, Germany

**Keywords:** *Arabidopsis thaliana*, auxin, cytokinin, hormonal cross-talk, lateral root development, root system architecture.

## Abstract

The plant root system is important for the uptake of water and nutrients and the anchoring of plants in the soil. Lateral roots (LRs) contribute considerably to root system architecture. Their post-embryonic formation is regulated by hormones and environmental cues. The hormone cytokinin influences LR formation and growth in *Arabidopsis thaliana* on different levels by disturbing cell division activity and pattern formation. This includes inhibition of the first formative cell division of pericycle founder cells and inhibition of the outgrowth of young LR primordia. Mutant analysis revealed that the cytokinin biosynthesis genes *IPT3* and *IPT5* and all three cytokinin receptor genes (*AHK2*, *AHK3*, and *CRE1/AHK4*) act redundantly during LR initiation. Mutation of *AHK2* and *AHK3* caused increased auxin sensitivity of LR formation, corroborating the functional relevance of auxin–cytokinin interaction during LR formation. In contrast, LR development of cytokinin receptor mutants in response to other hormones was mostly similar to that of the wild type, which is consistent with separate response pathways. A noticeable exception was an increased sensitivity of LR elongation to brassinolide in *ahk2 ahk3* mutants indicating antagonistic action of cytokinin and brassinosteroid. It is proposed that the multilevel redundancy of the cytokinin system in modulating LR formation reflects its role in mediating environmental cues.

## Introduction

The root system of dicotyledonous flowering plants comprises a primary root and lateral roots (LRs). LR development contributes considerably to the architecture of the root system and determines a substantial part of the ability of a plant to secure anchorage and extract micro- and macronutrients from the soil (Lynch, 1995). Unlike the primary root that originates during embryogenesis, LRs form throughout the life of the plant. They are formed from pericycle cells adjacent to the xylem poles, called pericycle founder cells in *Arabidopsis* (Casimiro *et al.*, 2001; Dubrovsky *et al.*, 2001; reviewed by Perét *et al.*, 2009; Benková and Bielach, 2010). These cells undergo a defined programme of oriented cell divisions and expansion to form a lateral root primordium (LRP). LRPs grow via both cell division and cell expansion, and then emerge from the parent root primarily by cell expansion. Once emerged, the LRP undergoes an activation step to form a fully functional LR meristem that directs the growth of LRs from this stage onwards (Malamy and Benfey, 1997).

The development of LRs is regulated coordinately by plant hormones and environmental signals. Numerous studies have shown that auxin plays a major role in LR development (reviewed in Péret *et al.*, 2009; Dastidar *et al.*, 2012). Auxin regulates several stages of LR development, initially in establishing a population of rapidly dividing pericycle cells and, later, the emergence of LRs (Himanen *et al.*, 2002). It has been reported that for proper LR initiation and LRP development, establishment of an auxin gradient with the maximum at the tip is important and the formation of a gradient mediated by PIN (PIN-FORMED) auxin efflux facilitators (Benková *et al.*, 2003; Geldner *et al.*, 2004). During LR initiation, the auxin accumulating in founder cells successively activates the auxin response modules SOLITARY ROOT (SLR)/INDOLE-3-ACETIC ACID (IAA)14, AUXIN RESPONSE FACTOR (ARF)7–ARF19, and BODENLOS/IAA12–MONOPTEROS/ARF5 to initiate the cell divisions leading to organogenesis (Fukaki *et al.*, 2002; Okushima *et al.*, 2005; De Smet *et al.*, 2010).

Several other hormones including brassinosteroid (BR), abscisic acid (ABA), and ethylene are involved in regulating LR formation (Fukaki and Tasaka, 2009). BR acts as a positive regulator of LR initiation by increasing acropetal auxin transport (Bao *et al.*, 2004) while ABA is a negative regulator of LR emergence (De Smet *et al.*, 2003). Ethylene affects LR development differently in a dose-dependent manner. Low concentrations of the ethylene precursor 1-aminocyclopropane-1-carboxylic acid (ACC) promote LR initiation, while higher doses strongly inhibit LRP initiation, but promote the emergence of existing LRPs (Ivanchenko *et al.*, 2008).

Another important hormonal regulator of root system architecture is cytokinin. The inhibitory effect of exogenous cytokinin on LR formation has been known for a long time (Torrey, 1959, 1962; Böttger, 1974). Convincing evidence that cytokinin has a physiologically relevant role during LR formation came from the analysis of plants with a lowered cytokinin status. A lowered cytokinin status was achieved by transgenic overexpression of *CKX* genes encoding cytokinin-degrading cytokinin oxidase/dehydrogenases (Werner *et al.*, 2001, 2003), by mutations in cytokinin biosynthetic isopentenyltransferase (*IPT*) genes (Miyawaki *et al.*, 2006), by cytokinin receptor mutation (Riefler *et al.*, 2006), or by abbreviating cytokinin signalling (Mason *et al.*, 2005; Yokoyama *et al.*, 2007; Heyl *et al.*, 2008; Ishida *et al.*, 2008). A low cytokinin status confined to the roots or early stages of LR formation enhances LR number (Laplaze *et al.*, 2007; Werner *et al.*, 2010). Relatively little is known about the mechanism of how cytokinin regulates LR development. Cytokinin inhibits LR initiation by blocking cell cycling in the pericycle via reduction of the expression of cyclin genes at the G_2_ to M transition (Li *et al.*, 2006). Cytokinin also interferes with the expression of *PIN* genes in LR founder cells, thereby preventing the establishment of an auxin gradient which is required for LR formation (Laplaze *et al.*, 2007). A post-transcriptional regulation by cytokinin of auxin action during LR organogenesis affects PIN1 trafficking and redirects it for lytic degradation in vacuoles (Marhavý *et al.*, 2011). It was demonstrated that cytokinin regulates LR organogenesis spatiotemporally and that initiation takes place in a root zone with elevated cytokinin levels but repressed cytokinin responses (Lohar *et al.*, 2004; Bielach *et al.*, 2012). Except for the cross-talk with auxin, little is known about the interactions of cytokinin with other hormones during LR formation. A transcriptomic analysis of cytokinin action in roots has identified numerous genes responding to the hormone with an altered transcript level, suggesting that this could be a functionally relevant level of regulation (Brenner *et al.*, 2012).

So far, most of the studies pertaining to the inhibitory role of cytokinin on LR formation are confined to the density of emerged LRs and largely neglected LRP formation. However, the density of emerged LRs is not necessarily useful to judge whether LR formation is affected in a genotype or by a treatment (De Smet *et al.*, 2012; Dubrovsky and Forde, 2012). This is because LR emergence is highly susceptible to genetic and environmental factors that are distinct from those affecting LR initiation. The effects of exogenously added cytokinin or *in vivo* lowered cytokinin levels on both LRP and LR formation was therefore analysed here in detail. This work has confirmed a role for cytokinin early during LR formation and revealed a large degree of functional redundancy among cytokinin metabolism and signalling genes. It is shown that the cytokinin receptors AHK2 and AHK3 are involved in the interaction of cytokinin with auxin during LR formation. Investigations involving other hormones yielded only little support for cross-talk with cytokinin but indicated mainly the existence of separate pathways.

## Materials and methods

### Plant material and growth conditions

All *Arabidopsis thaliana* plants used in this study were of the Columbia (Col-0) ecotype. Seeds of the transgenic line *CycB1;1:GUS* (Beeckman *et al.*, 2001) and *ipt* mutants (Miyawaki *et al.*, 2006) were kindly provided by Dr Laurent Laplaze and Dr Tatsuo Kakimoto, respectively. The transgenic line *35S:CKX1* and the cytokinin receptor double mutants *ahk2 ahk3*, *ahk2 cre1*, and *ahk3 cre1* were described previously (Werner *et al.*, 2003; Riefler *et al.*, 2006). Seeds were surface-sterilized with 1.2% sodium hypochlorite and 0.01% Triton X-100 and sown on sterile plates containing half-strength Murashige and Skoog (MS) medium supplemented with 1% sucrose and 0.9% agar. The plates were sealed and seeds were placed at 4 °C for 3 d in the dark prior to germination. After stratification treatment, all plates were placed vertically under white light (~100 μmol m^–2^ s^–1^) in long-day conditions (16h light/8h darkness) at 22 °C.

### Histochemical analysis

Histochemical staining for β-glucuronidase (GUS) activity was performed according to Jefferson *et al.* (1987). Briefly, seedlings were immersed in a staining solution [100mM phosphate, pH 7.0, 10mM EDTA, 0.5mM K_3_Fe(CN)_6_, 0.5mM K_4_Fe(CN)_6_·3H_2_O, 1ml l^–1^ Triton X-100, 0.5mg ml^–1^ X-Gluc] at 37 °C after a brief vacuum treatment. After staining, seedlings were cleared and mounted according to Malamy and Benfey (1997). The GUS staining pattern was recorded with a Zeiss Axioskop microscope.

### Analysis of root architecture traits

Primary root length and LR length were measured on digital images of the plates using ImageJ 1.40 software (http://rsb.info.nih.gov/ij/). The number of emerged LRs was counted using a binocular. The number of LRPs was determined using a Zeiss Axioskop microscope. The clearing of tissues and classification of LRP developmental stages were performed according to Malamy and Benfey (1997). Data were analysed using Excel^®^. Experiments were repeated at least twice independently.

### Hormone treatments

Regarding cytokinin treatment, seedlings were germinated and grown on medium with different concentrations of 6-benzylaminopurine (BA) for 11 d. BA was dissolved in 1 N sodium hydroxide (NaOH) to make a stock solution. Seedlings were germinated on hormone-free medium and transferred after 4 d to fresh half-strength MS medium containing various concentrations of these hormones and grown for another 7 d for the determination of the sensitivity of root growth and LR formation to the hormones 1-naphthaleneacetic acid (NAA), ABA, brassinolide (BL), and ACC. Concentrated stocks of 1mM NAA in 1 N NaOH, BL in dimethylsulphoxide (DMSO), and ACC in water were added to agar medium cooled to 50 °C after autoclaving. ABA dissolved in ethanol was added to the medium at the specified concentrations before autoclaving.

## Results

### Cytokinin inhibits LR formation through inhibition of cell division and pattern formation

**Fig. 1.**
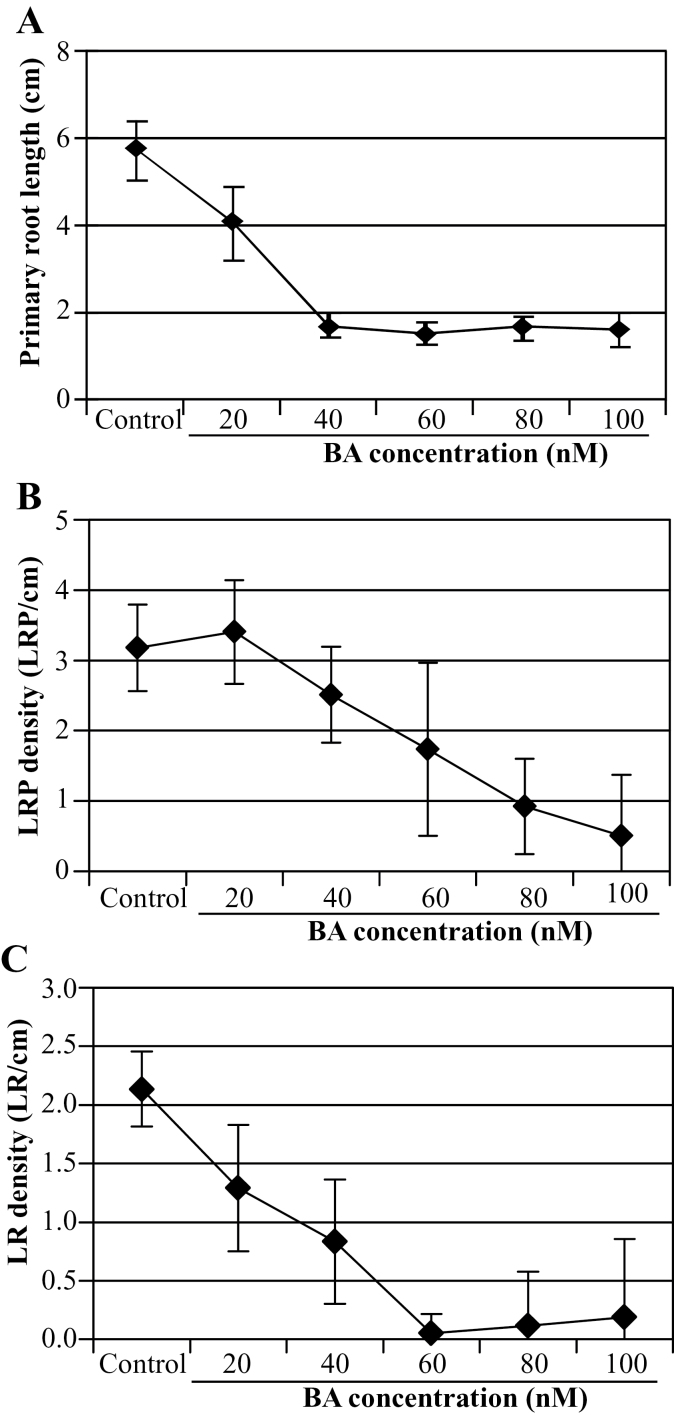
Exogenous cytokinin inhibits primary root elongation and LR formation. The inhibitory action of cytokinin on (A) primary root growth, (B) LRP density, and (C) LR density is shown. *CycB1;1:GUS* seedlings were grown on vertical agar plates supplemented with 0, 20, 40, 60, 80, and 100nM BA. The numbers of LRPs and emerged LRs were counted 10 d after germination. Error bars indicate ±SD. *n*=25 in (A, C), *n*=15 in (B).

**Fig. 2.**
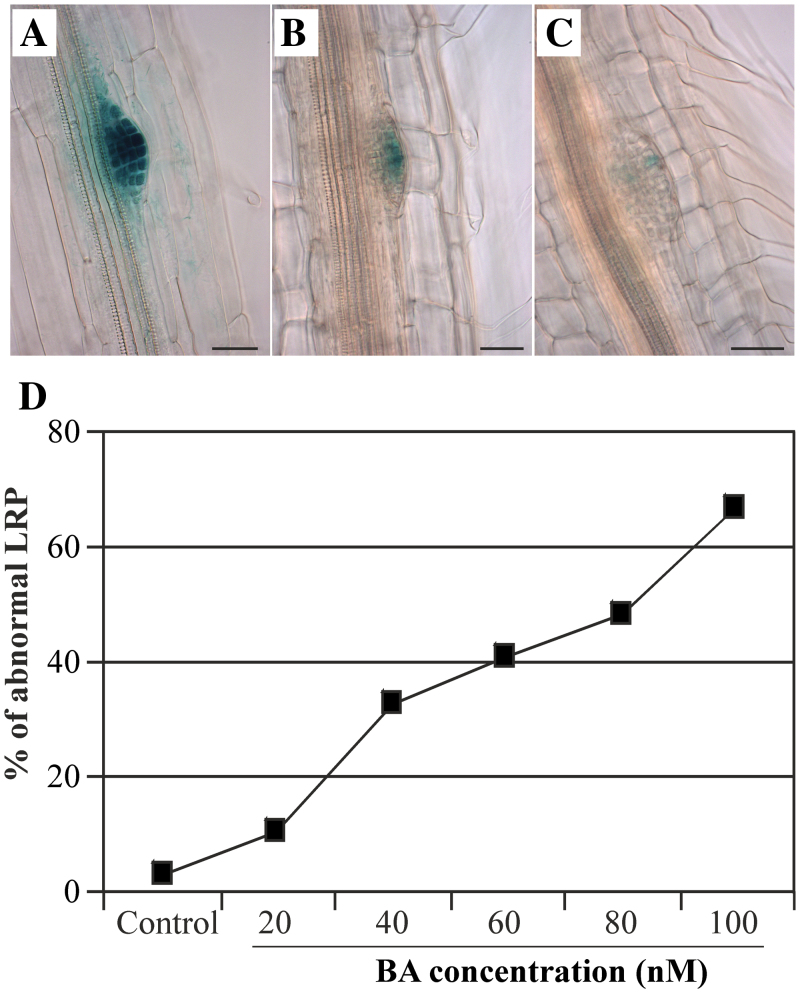
Exogenous cytokinin perturbs the organization of LRPs and *CycB1;1:GUS* expression. Seedlings were grown on medium supplemented with (A) 0nM, (B) 40nM, and (C) 80nM BA, respectively. LRP organization and GUS staining were evaluated 11 d after sowing. Increasing BA concentrations caused earlier growth arrest, lower *CycB1;1:GUS* expression, and more severe patterning defects. Bar=20 μM. (D) The proportion of abnormal LRPs formed on medium supplemented with different concentrations of BA.

In order to investigate how cytokinin affects root development and cellular pattern formation during LR development under the experimental conditions used here, seeds of a transgenic *Arabidopsis* line containing a reporter gene consisting of the *CycB1;1* gene promoter and the *GUS* gene (*CycB1;1:GUS*) were germinated and grown on media containing different cytokinin (BA) concentrations. *CycB1;1:GUS* was reported to be a good marker to visualize the site of LRP initiation and development (Beeckman *et al.*, 2001). Seedling roots were cleared and the primary root length, LRP density (number of LRP cm^–1^ primary root), LR density (number of emerged LR cm^–1^ primary root), and the organization of LRPs were analysed (Figs 1, 2).

Figure 1 shows that primary root length, and LRP and LR density decreased with increasing cytokinin concentration, confirming the inhibitory role of the hormone in primary root elongation and LR development. Primary root growth and LR outgrowth were arrested almost completely by 40nM and 60nM BA, respectively (Fig. 1A, C). In contrast, the density of LRPs at these cytokinin concentrations was lowered to only about half of the value on hormone-free control medium (Fig. 1B). This shows that the LR initiation process is less sensitive than LR emergence to cytokinin.

The division of cells and their organization in LRPs after cytokinin treatment were analysed in more detail in the *CycB1;1:GUS* transgenic line (Fig. 2). Formation of LRPs showing a disorganized cellular pattern was observed with the increase in cytokinin concentrations, indicating abnormal early cell division during LRP formation (Fig. 2A–C). The proportion of abnormal LRPs increased with increasing cytokinin concentration (Fig. 2D). In plants treated with 100nM BA, 66.7% of the LRPs were disorganized, but this was so for only 3.1% of the plants grown on hormone-free medium (Fig. 2D). At the same time, the number of cells undergoing division was strongly reduced, as is indicated by the lower number of cells showing blue GUS staining which marks dividing cells (Fig. 2). This is in agreement with a previous study (Laplaze *et al.*, 2007). The perturbation of cell division and formation of the correct cellular pattern in early stages of LRP development is possibly contributing to their failure to emerge and form a mature LR.

### Cytokinin deficiency promotes LR initiation

LR formation was analysed in a number of cytokinin-deficient transgenic plants and mutants in order to explore the effect of a lowered cytokinin status on LR formation and development, and the involvement of different cytokinin metabolism and signalling genes. These included plants with a reduced cytokinin content because of an enhanced breakdown (*35S:CKX1*; Werner *et al.*, 2003) or a reduced synthesis (*ipt* mutants; Miyawaki *et al.*, 2006), and cytokinin receptor mutants (Riefler *et al.*, 2006). The developmental stage of each LRP was classified according to Malamy and Benfey (1997).

**Fig. 3.**
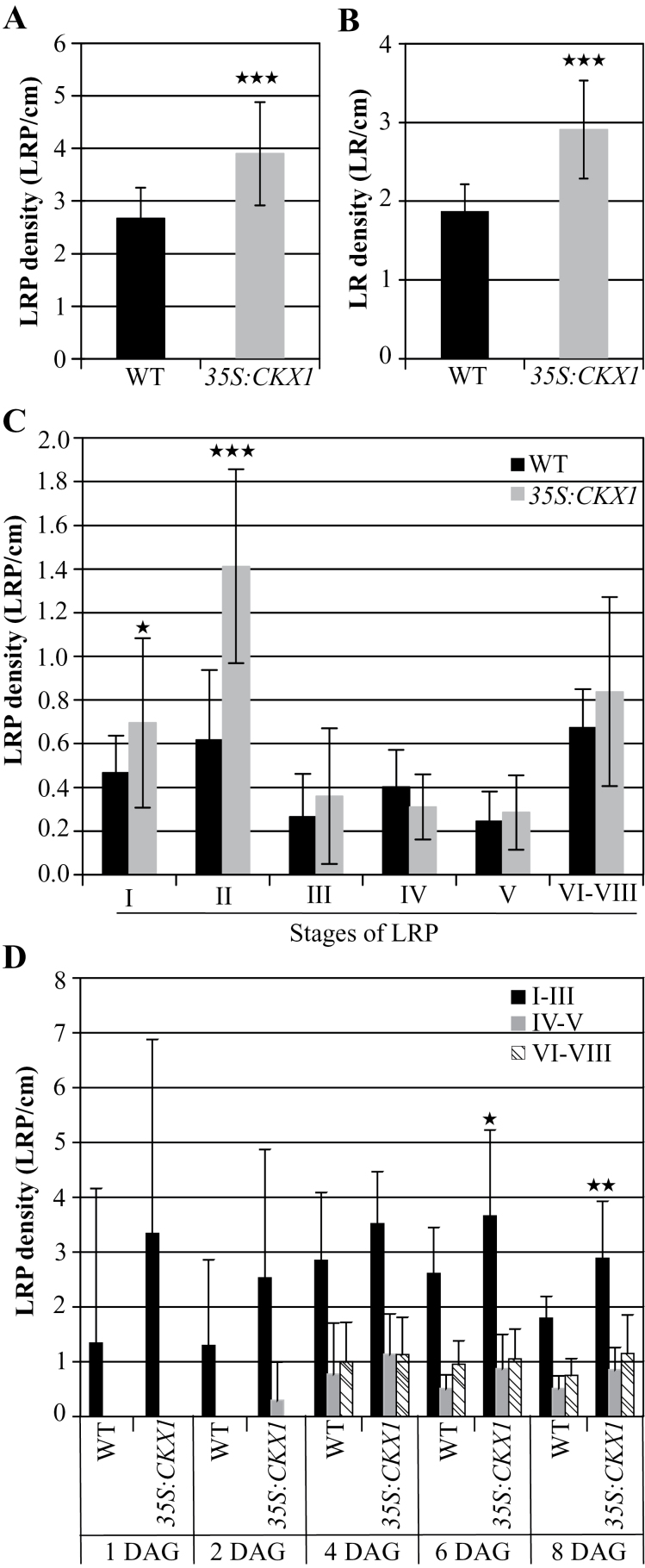
LR formation in cytokinin-deficient *35S:CKX1* transgenic seedlings. *35S:CKX1* plants showed an increased density of (A) LRPs and (B) LRs compared with the wild type. (C) Density of LRPs in different developmental stages. *35S:CKX1* plants have more LRPs in early stages than the wild type. (D) LRP density of *35S:CKX1* transgenic seedlings compared with wild-type seedlings at different time points after germination. LRP formation and development occurred earlier in *35S:CKX1* transgenic seedlings. The number of LRPs and emerged LRs were counted 10 d after germination (DAG) in (A–C) and as indicated in (D). The values shown are means ±SD. *n* ≥15. Significance of differences was analysed by Student’s *t*-test. *P < 0.05, **P < 0.01, ***P < 0.001.

The *35S:CKX1* seedlings showed a strong increase of LRP (46%) and LR density (56%) compared with the control seedlings (Fig. 3A, B). The morphological analysis revealed a higher number of stage I and stage II primordia, with a >2-fold increase in the latter (Fig. 3C). Analysis of LRP formation of *35S:CKX1* seedlings at different time points after germination showed that these also produce more LR than the wild type at earlier stages, with an increasing number of LRPs of stage I–III from 6 d after germination onwards (Fig. 3D). Together, this indicates that cytokinin deficiency promotes LR initiation in particular.

**Fig. 4.**
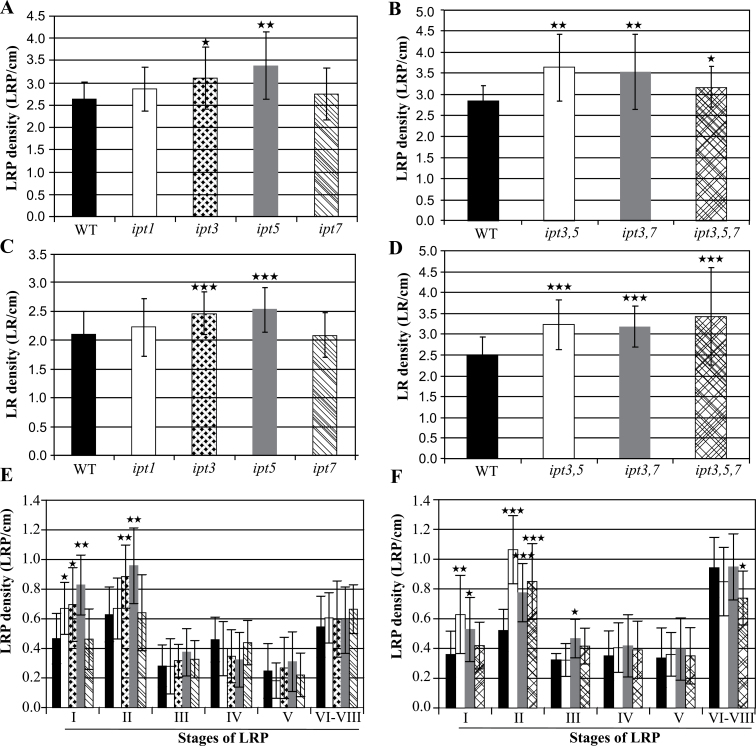
LR formation in *ipt* mutant seedlings. LRP (A) and LR (C) density of single *ipt* gene mutants. LRP (B) and LR (D) density of different *ipt* double and triple mutants. (E, F) LRPs of different stages formed on the primary roots of *ipt* transgenic seedlings compared with wild-type seedlings. The filling of bars corresponds in (E) to the same genotypes as in (A) and (C), and in (F) to the same genotypes as in (B) and (D). The number of LRPs and emerged LRs was counted 10 d after germination. The values shown are means ±SD. *n* ≥15. Significance of differences was analysed by Student’s *t*-test. *P < 0.05; **P < 0.01; ***P < 0.001.

Next experiments were carried out to determine which cytokinin synthesis (*IPT*) genes are involved in LR initiation. Miyawaki *et al.* (2006) reported that the *Arabidopsis ipt3 5 7* triple and the *ipt1 3 5 7* quadruple mutants have slightly longer primary roots and more LRs longer than 1cm compared with the wild type. Here, the LR formation of *ipt* single, double, and triple mutants was examined in more detail (Fig. 4). Only *ipt3* and *ipt5* among the single mutants showed an increased LRP and LR density, while *ipt1* and *ipt7* behaved similarly to the wild type (Fig. 4A, C). The enhancement of total LRP density of the *ipt3* and *ipt5* mutants was mainly due to an increase of the stage I and stage II primordia, similar to the *35S:CKX1* seedlings (Fig. 4E). The double and triple mutants *ipt3 5*, *ipt3 7*, and *ipt3 5 7* also showed a strongly increased LRP and LR density compared with the wild type (Fig. 4B, D) and an enhanced density of early LRP developmental stages (Fig. 4F). These results proved that a lowered cytokinin biosynthesis promotes LR initiation and LR development, thus further supporting the physiological role of cytokinin in this process. In addition, the *IPT3* and *IPT5* genes are particularly relevant to regulate LR initiation.

### Redundant and specific actions of cytokinin receptor genes in regulating LR formation

**Fig. 5.**
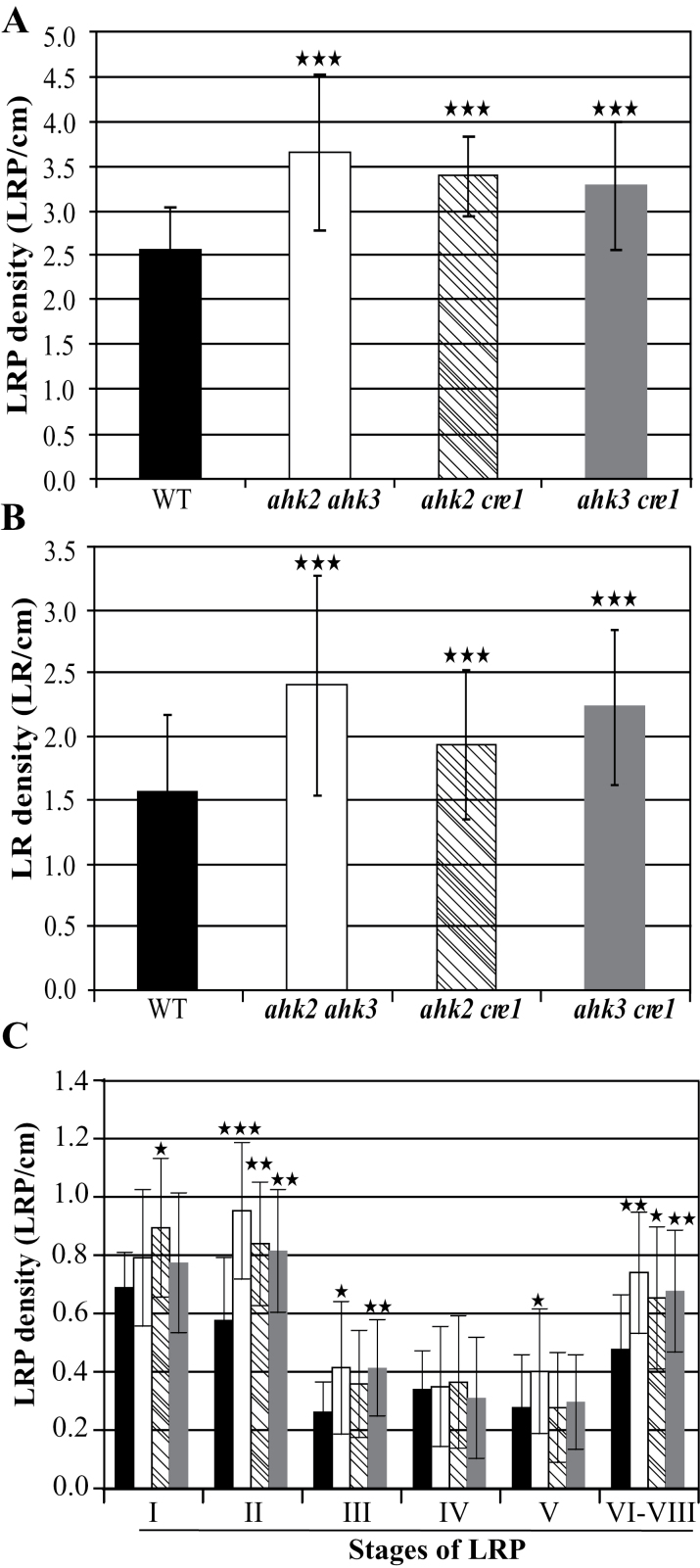
LR formation in cytokinin receptor mutants. LRP (A) and LR (B) density in cytokinin receptor mutants compared with the wild type. (C) LRPs of different stages formed on the primary roots of the three double receptor mutants compared with the wild type. The filling of bars in (C) corresponds to the same genotypes as in (A) and (B). The numbers of LRPs and emerged LRs were counted 10 d after germination. The values shown are means ± SD. *n* ≥15. Significance of differences was analysed by Student’s *t*-test. *P < 0.05; **P < 0.01; ***P < 0.001.

In order to explore which of the three cytokinin receptors would be involved in regulating LR formation, LRP and LR density were measured in the three double cytokinin receptor mutants *ahk2 ahk3*, *ahk2 cre1*, and *ahk3 cre1*, each of them retaining a different single cytokinin receptor. All three mutants had a higher LRP and LR density compared with the wild type, indicating a high degree of functional redundancy of the three receptors in regulating LR development (Fig. 5). The LRP densities of all three double receptor mutants were increased significantly in both early (in particular stage II) and late (VI–VIII) stages of the primordium, while they were comparable with the wild type in the intermediate stages (Fig. 5C). This suggested that cytokinin receptors act not only during the LR initiation, but also during the emergence of LRs.

### Expression of a cytokinin reporter gene during LR development

**Fig. 6.**
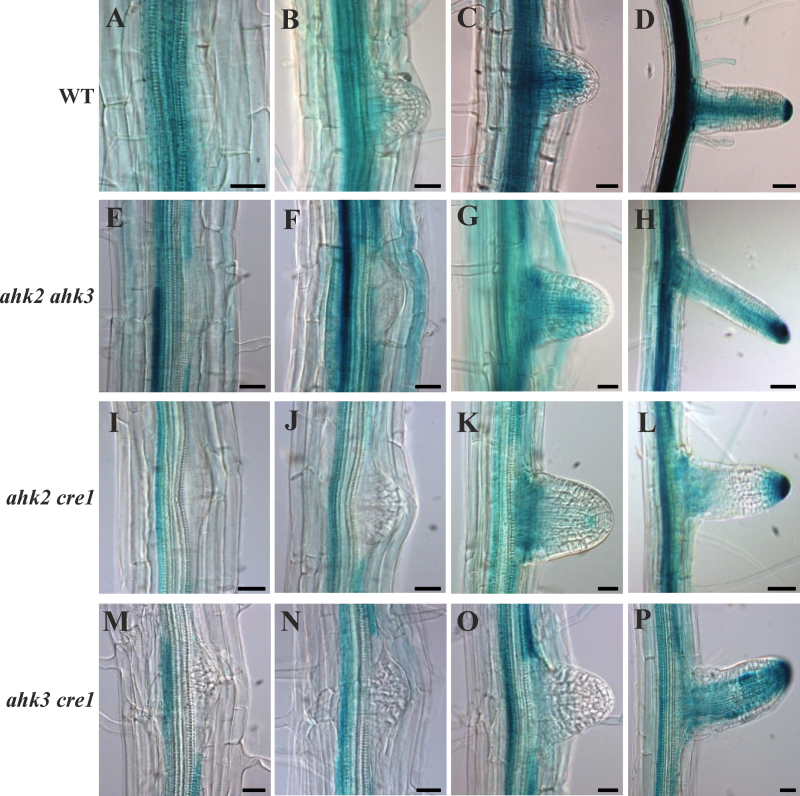
Expression of *ARR5:GUS* in the wild type and cytokinin receptor mutants during LR formation. *ARR5:GUS* expression is indicated by blue staining in LRs of different stages (from left to right: early, late, emerged, mature) in the wild type (A–D), and *ahk2 ahk3* (E–H), *ahk2 cre1* (I–L), and *ahk3 cre1* (M–P) mutants. Eleven-day-old *Arabidopsis* seedlings were incubated in staining solution for 6h. Typical staining patterns are shown for each genotype and stage (*n*=20). Bar in (A–C, E–G, I–K, M–P)=20 μM; bar in (D, H, L)=50 μM.

The cytokinin reporter gene *ARR5:GUS* (D’Agostino *et al.*, 2000) was introgressed into the mutant background (Stolz *et al.*, 2011) and its expression pattern compared between the wild type and the three double receptor mutants to study the dynamics and tissue specificity of the cellular cytokinin status changes during LR development (Fig. 6). *ARR5:GUS* in wild-type roots was expressed in the vascular bundle of the primary root and no expression was detected in the early stage of LRPs (Fig. 6A). This pattern is consistent with the one shown by the artificial cytokinin reporter gene *pTCSn:GFP* (Bielach *et al.*, 2012; Zürcher *et al.*, 2013), suggesting that the *ARR5:GUS* reporter used here records principally the cytokinin signal generated by the two-component signalling system. The expression of the *ARR5:GUS* reporter was much lower, particularly in the *ahk2 cre1* and *ahk3 cre1* background, in accordance with with the known prevalent role of CRE1/AHK4 in mediating the cytokinin response in the root vasculature (Fig. 6I, M) (Mähönen *et al.*, 2000, 2006). Only a weak expression was observed in the wild type at the base of the emerging LRP (Fig. 6B). Once the LRP emerged, strong GUS activity was detected in the newly formed LR vasculature (Fig. 6C, D). The expression of *ARR5:GUS* was absent in the non-emerged LRs of all three receptor double mutants (Fig. 6F, J, N). After emergence, the *ARR5:GUS* signal appeared at the base of the LRs in *ahk2 ahk3* and *ahk2 cre1* (Fig. 6G, K), but not in *ahk3 cre1* (Fig. 6O). The establishment of a cytokinin signal in the vasculature of the emerged LR required the presence of CRE1/AHK4, while each of the three receptors was sufficient to establish the signal in the LR tip (Fig. 6H, L, P).

### Cross-talk of cytokinin with auxin, BR, ABA, and ethylene during LR development

**Fig. 7.**
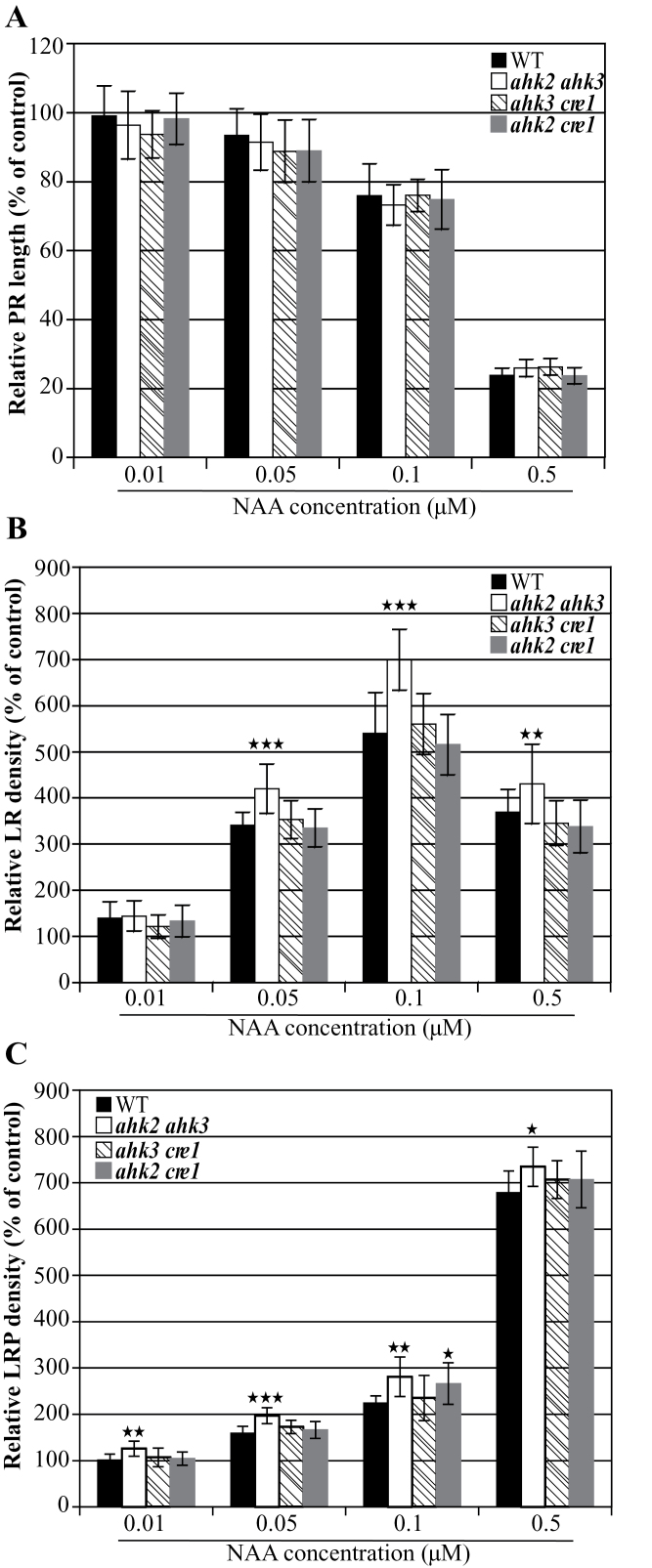
Root development of cytokinin receptor mutants in response to auxin. (A) Primary root (PR) growth, (B) LR density, and (C) LRP density measured on NAA-containing medium were expressed relative to data obtained on hormone-free medium. The values shown are means ±SD. *n*=20–36 in (A, B), *n*=10 in (C). Significance of differences was analysed by Student’s *t*-test. *P < 0.05; **P < 0.01; ***P < 0.001. WT, wild type.

It is well known that cytokinin modulates auxin action during LR formation (Laplaze *et al.*, 2007; Marhavý *et al.*, 2011); however, the relevance of the different cytokinin receptors has not yet been studied. Therefore, the response of the double receptor mutants to exogenous auxin was investigated and compared (Fig. 7). The inhibition of primary root elongation by NAA was similar in the wild type and all three receptor double mutants (Fig. 7A). In contrast, LR and LRP density of the *ahk2 ahk3* mutant was increased more strongly than in the wild type in response to NAA treatment. For example, *ahk2 ahk3* mutant seedlings formed ~30% and 25% more LR and LRP, respectively, than the wild type on medium containing 0.1 μM NAA (Fig. 7B, C). These results suggest that a reduction of cytokinin signalling increases the sensitivity to auxin during LR development, and that AHK2 and AHK3 are mainly involved in mediating the action of cytokinin in the cross-talk with auxin during LR formation.

**Fig. 8.**
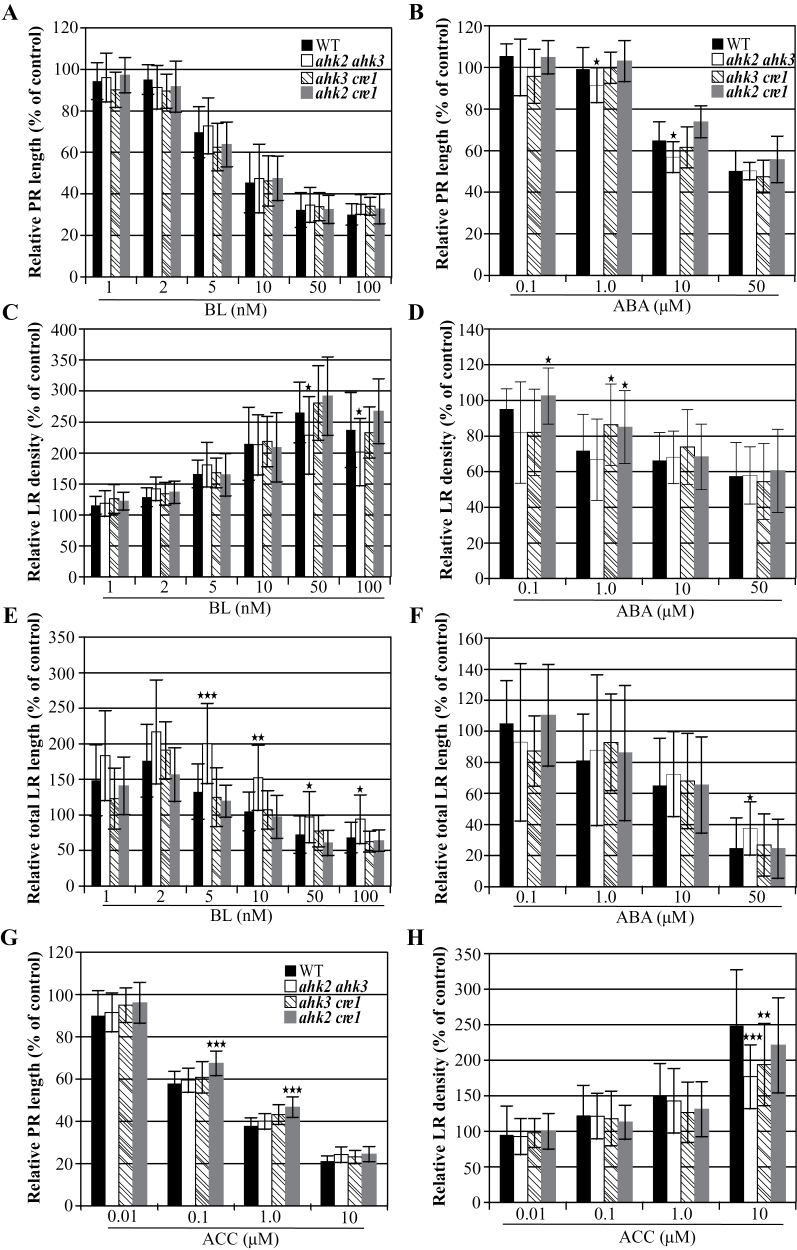
Root development of cytokinin receptor mutants in response to different plant hormones. The length of the primary root, LR density, and total length of LRs in response to BL (A, C, E), ABA (B, D, F), and ACC (G, H) were compared between the wild type and double cytokinin receptor mutants. Data are mean values ±SD. *n* ≥24 for primary root length and LR density; *n* ≥15 for total LR length. Asterisks indicate statistically significant differences from the wild type determined using Student’s *t*-test. * *P < 0.05; **P < 0.01; ***P < 0.001. PR, primary root; WT, wild type.

Next, it was investigated whether the response to other hormones regulating root elongation and branching was also altered as a consequence of cytokinin receptor gene mutation. Because cytokinin, BR, and ABA also affect LR elongation (De Smet *et al.*, 2003), this parameter was additionally included in the cross-talk analysis. Figure 8A shows that the primary root length of the wild type and the receptor mutants decreased similarly after BL treatment. LR density of all genotypes also responded similarly to BL, with a bit less increase of the LR density in *ahk2 ahk3* mutants on 50nM and 100nM BL (Fig. 8C). Low concentrations of BL increased LR growth in the wild type and all cytokinin receptor mutants. However, a higher increase of LR growth was found in *ahk2 ahk3* mutants (Fig. 8E). This is consistent with a synergistic action of BL and a reduced cytokinin content on LR growth (Vercruyssen *et al.*, 2011), and indicates a role for AHK2 and AHK3 in this BL–cytokinin cross-talk.

The sensitivity of all three cytokinin receptor mutants to ABA was comparable to that of the wild type with respect to primary root elongation, LR density, and LR elongation, except for a slightly higher resistance of *ahk2 cre1* and *ahk3 cre1* on 1 μM ABA (Fig. 8B, D, F). Together this suggests that cytokinin and ABA pathways acting on LR development are mostly separate. The inhibitory effects of ACC on primary root elongation of *ahk2 ahk3* and *ahk3 cre1* were also similar to those of the wild type, but *ahk2 cre1* mutants showed higher resistance than the wild type on medium containing 0.1 μM and 1.0 μM ACC (Fig. 8G). This indicates that part of the inhibitory effect of ACC on primary root elongation is mediated through cytokinin via AHK2 and CRE1/AHK4. The LR density of the wild type increased after the application of different concentrations of ACC, and all three double receptor mutants responded in a similar fashion at low ACC concentrations (0.01–1.0 μM). A significantly lower increase of LR density was observed in *ahk2 ahk3* and *ahk3 cre1* at the highest ACC concentration (10 μM) (Fig. 8H). It is inferred from this result that AHK3 is required to mediate the stimulating effect of high concentrations of ACC on LR development.

## Discussion

### The inhibitory effect of cytokinin on LR initiation and LR development

The dose–response curves of inhibition of LRP formation and outgrowth of LRs by cytokinin differed. LRPs were still formed at low frequency on media with cytokinin concentrations that inhibited the outgrowth of these primordia completely (Fig. 1). High cytokinin concentration inhibited the first cell divisions of the pericycle founder cells in agreement with Laplaze *et al.* (2007). Early cytokinin action in xylem pole pericycle cells blocks LR initiation owing to an arrested G_2_–M transition (Li *et al.*, 2006). Extended analysis of the spatiotemporal action of cytokinin using tissue- and stage-specific promoters driving an *IPT* gene suggested that cytokinin might act even prior to the first division of periclinal cells to inhibit LR formation (Bielach *et al.*, 2012). Fine-tuning of early action of cytokinin is demonstrated by the functioning of AHP6, a negative regulator of the cytokinin signalling pathway, in pericycle cell division (Moreira *et al.*, 2013). It was noted at intermediate cytokinin concentrations, which were not completely inhibitory for LR initiation, that an increase in cell cycle inhibition in LRPs of later stages occurred, indicating that the cell cycle is a target of cytokinin action during LR emergence (Fig. 2). Cytokinin caused the formation of a disorganized pattern in the emerging LR (mainly in stages IV and V), similar to those described by Laplaze *et al.* (2007). This cytokinin response of LRPs is in contrast to the absence of a visible cytokinin reporter gene signal in the wild type and all cytokinin receptor mutants at this stage. Indeed, no noticeable cytokinin signal was present from the stage of LR initiation until LR emergence (Fig. 6; Bielach *et al.*, 2012; Zürcher *et al.*, 2013). Strong cytokinin receptor expression and activity were only re-established at advanced stages and mimicked the pattern seen in the primary root (Nishimura *et al.*, 2004; Stolz *et al.*, 2011). This suggests that cytokinin signalling is turned down during the emergence phase of the LR. Moreover, it is noteworthy that compared with an endogenous cytokinin concentration in the low nanomolar range in roots (Werner *et al.*, 2010) and a saturation of cytokinin receptor action (measured as reporter gene activity) at ~20nM exogenously added *trans*-zeatin (Stolz *et al.*, 2011), relatively high cytokinin concentrations (>50nM BA) were required here to cause strong cell cycle inhibition and altered cellular pattern in LRPs. These experiments are not informative about the locally active cytokinin concentrations in the root but they do not lend strong support for cell cycle inhibition being a physiologically relevant cytokinin effect pathway during LR development.

### Redundancy of the cytokinin system regulating root system architecture

Mutant analysis identified cytokinin metabolism and signalling genes involved in regulating LR formation. Among the cytokinin-synthesizing genes, *IPT3* and *IPT5* genes were recognized as particularly relevant, while *IPT1* and *IPT7* appear to play no role in LR formation. LRPs of stages I and II were over-represented in *ipt3* and *ipt5* single and double mutants (Fig. 4), suggesting the action of cytokinin during this early stage. *IPT3* is expressed in the phloem and pericycle, as well as in the basal stele of young LRs. *IPT5* expression was detected in the pericycle, LRP, and the columella of emerging LRs (Miyawaki *et al.*, 2004; Takei *et al.*, 2004). Thus, their site of expression is consistent with their site of action. It should be noted that the cytokinin-activating *LOG3* and *LOG4* genes (Kuroha *et al.*, 2009) and the cytokinin-degrading *CKX5* and *CKX6* genes are expressed at the same stage (Werner *et al.*, 2003), indicating a fine-tuned balance of cytokinin synthesis and breakdown in young LRPs.

LR formation in all three double receptor mutants was enhanced, suggesting that all three receptors are involved in its regulation (Fig. 5). Again, early developmental stages were abundant, consistent with a function for the receptor in the initial stages of LR development. In addition, the higher frequency of LRPs of late stages (VI–VIII) pointed to a function for cytokinin receptors after root emergence and meristem establishment, which is in accordance with the onset of their signalling activity (Fig. 6). Functional redundancy in regulating LR formation was also found among the type-B *ARR* genes (*ARR1*, *ARR10*, and *ARR12*) that operate downstream of the receptors (Mason *et al.*, 2005). In contrast, Zhang and colleagues reported that even octuple type-A *ARR* gene mutants show an LR density similar to that of the wild type, indicating a limited role for this gene family (Zhang *et al.*, 2011). It is interesting that mutation of type-A *ARR* genes had a stronger negative effect on the formation of second-order LRs, suggesting that their formation might be more cytokinin sensitive and/or that different regulatory circuits operate during the formation of primary and secondary LRs (Zhang *et al.*, 2011).

Together, the consequences of gene mutation and the regulation of cytokinin metabolism and signalling genes demonstrate that both the control of hormone concentration and signal perception and transduction are important to realize its physiological role during LR formation. The number of cytokinin genes involved and their apparent functional redundancies leads to the proposal that cytokinin acts as an integrator of different environmental information acting through different branches of the cytokinin system and impinging on LR development. Indeed, cytokinin has a role in mediating the response to various nutritional cues known as regulators of LR formation, including the availability of phosphorus, sulphur, nitrogen, and iron (reviewed by Argueso *et al.*, 2009; Rubio *et al.*, 2009; Werner and Schmülling, 2009), and the transcript level of nutrient transporter genes in the roots depends on the cytokinin status (Werner *et al.*, 2010; Brenner *et al.*, 2012).

### Cross-talk of cytokinin and auxin signalling pathways

It is known that cytokinin and auxin play antagonistic roles during LR organogenesis, and different mechanisms underlying their antagonistic activities have been revealed. Exogenous cytokinin represses the expression of *PIN* genes which encode auxin efflux carriers, and thus perturbs the establishment of an auxin gradient needed for re-specification of founder cells during LR initiation (Laplaze *et al.*, 2007). Perturbation of auxin gradient formation will also be the consequence of enhanced PIN1 degradation triggered by cytokinin in LRPs (Marhavý *et al.*, 2011). Transcriptomic analyses have shown that auxin fine-tunes cytokinin action by regulating metabolism and signalling genes. For example, a strong up-regulation of the *CKX1* and, in particular, *CKX6* gene (Werner *et al.*, 2006; Paponov *et al.*, 2008) and a mostly down-regulation of receptor (*AHK3* and *AHK4*) and several transcription factor genes (e.g. *ARR11*) during the asymmetric division of xylem pole pericycle cells (Parizot *et al.*, 2010) indicate a lowered cytokinin status in response to auxin action. The regulation of most of the cytokinin genes by auxin depends at least partly on the two successive auxin response modules SLR/IAA14 and ARF7/ARF19 (Parizot *et al.*, 2010), indicating that they are downstream components of the auxin LR initiation pathway. Noticeable exceptions were the *IPT3* and *CKX1* genes, which responded independently of this pathway (Parizot *et al.*, 2010).

A functional link between cytokinin and auxin action is corroborated by the stronger increase of LR and LRP density in the *ahk2 ahk3* receptor double mutant in response to auxin compared with the wild type (Fig. 7B, C). This reveals that cytokinin acting through these two receptors normally counterbalances auxin action and that this interaction between the cytokinin and auxin pathways takes place early during LR formation. It is interesting that mutation of *CRE1/AHK4* did not change the response to auxin, although this receptor is required for cytokinin-stimulated PIN1 degradation (Marhavý *et al.*, 2011).

### Cross-talk of cytokinin with other hormones during LR development

Studying the consequences of cytokinin receptor gene mutations for the action of BR, ABA, and ethylene on primary root elongation, LR density, and LR elongation has yielded only limited indications for cross-talk between cytokinin and these other hormones (Fig. 8). Inhibition of primary root growth and the increase of LR density by BL was similar in the wild type and receptor mutants. This is consistent with separate pathways for BR and cytokinin, in agreement with previous results (Vercruyssen *et al.*, 2011). However, *ahk2 ahk3* responded more sensitively than the wild type, *ahk3 cre1*, and *ahk2 cre1* to the stimulation of LR elongation by low concentrations of BL. This shows that cytokinin antagonizes the stimulatory BR action on LR elongation acting through AHK2 and AHK3. Consistently, stimulation of LR elongation by BL was stronger in plants with a lowered cytokinin content than in the wild type (Vercruyssen *et al.*, 2011).

Cytokinin receptor mutants displayed only a weak resistance to LR inhibition by low concentrations of ABA. This limited interaction of cytokinin and ABA pathways could be due to the timing of the ABA-induced suppression of LR development, which occurs post-emergence of the LRP from the main root and prior to LR meristem activation. A key regulator is the *ABI4* (*ABA INSENSITIVE4*) gene, encoding an AP2 transcription factor, which is induced by ABA and cytokinin, and repressed by auxin in the root (Shkolnik-Inbar and Bar-Zvi, 2010). The present data (Fig. 8D) suggest that CRE1/AHK4 is required for the full activity of ABA at low concentrations.

A limited, concentration-dependent interaction was also found for ethylene and cytokinin. A low but significant influence of cytokinin receptor mutation (*ahk2 cre1*) was found on primary root elongation (Fig. 8G) at intermediate ACC concentrations. Similarly, LR formation of cytokinin receptor mutants (*ahk2 ahk3* and *ahk3 cre1*) was less sensitive to the stimulatory action of ACC only at the highest concentration that was tested (10 μM), while it was similar to the wild type at all lower concentrations.

### Conclusions

This and other work indicates that cytokinin constitutes one of several circuits, including other hormonal, mechanical, and osmotic signals, modulating LR development (Malamy, 2005; Werner and Schmülling, 2009). Cytokinin acts on specific stages of LR development, and the early stages of LRP formation are particularly cytokinin sensitive. Furthermore, it appears that cytokinin might have a second role; namely, to serve as a positional signal for the formation of new LRPs. Cytokinin metabolism and signalling genes form a redundant network to modulate LR formation and growth. That is consistent with a role for cytokinin as a mediator with the many environmental cues that regulate root system architecture. These cues are proposed to operate through fine-tuned transcriptional regulation of cytokinin metabolism and signalling genes to modulate LR development.

## Abbreviations:

ABA: abscisic acid
ACC: 1-aminocyclopropane-1-carboxylic acid
BA: 6-benzylaminopurine
BL: brassinolide
BR: brassinosteroid
LR: lateral root
LRP: lateral root primordium
NAA: 1-naphthaleneacetic acid.
